# Complex and unexpected outcomes of antibiotic therapy against a polymicrobial infection

**DOI:** 10.1038/s41396-022-01252-5

**Published:** 2022-05-21

**Authors:** Lydia-Ann J. Ghuneim, Ruma Raghuvanshi, Kerri A. Neugebauer, Douglas V. Guzior, Martin H. Christian, Bella Schena, Jeremiah M. Feiner, Alicia Castillo-Bahena, Jenna Mielke, Marc McClelland, Douglas Conrad, Isaac Klapper, Tianyu Zhang, Robert A. Quinn

**Affiliations:** 1 Department of Biochemistry and Molecular Biology, Michigan State University, East Lansing, MI 48824 USA; 2 Paris-Saclay University, INRAE, AgroParisTech, Micalis Institute, Jouy-en-Josas, France; 3 Department of Microbiology and Molecular Genetics, Michigan State University, East Lansing, MI 48824 USA; 4 Spectrum Health West Michigan, Grand Rapids, MI 49503 USA; 5 Department of Medicine, University of California San Diego, La Jolla, CA 92093 USA; 6 Department of Mathematics, Temple University, Philadelphia, PA 19122 USA; 7 Department of Mathematical Sciences, Montana State University, Bozeman, MT 59717 USA

**Keywords:** Microbiome, Microbial ecology

## Abstract

Antibiotics are our primary approach to treating complex infections, yet we have a poor understanding of how these drugs affect microbial communities. To better understand antimicrobial effects on host-associated microbial communities we treated cultured sputum microbiomes from people with cystic fibrosis (pwCF, *n* = 24) with 11 different antibiotics, supported by theoretical and mathematical modeling-based predictions in a mucus-plugged bronchiole microcosm. Treatment outcomes we identified in vitro that were predicted in silico were: 1) community death, 2) community resistance, 3) pathogen killing, and 4) fermenter killing. However, two outcomes that were not predicted when antibiotics were applied were 5) community profile shifts with little change in total bacterial load (TBL), and 6) increases in TBL. The latter outcome was observed in 17.8% of samples with a TBL increase of greater than 20% and 6.8% of samples with an increase greater than 40%, demonstrating significant increases in community carrying capacity in the presence of an antibiotic. An iteration of the mathematical model showed that TBL increase was due to antibiotic-mediated release of pH-dependent inhibition of pathogens by anaerobe fermentation. These dynamics were verified in vitro when killing of fermenters resulted in a higher community carrying capacity compared to a no antibiotic control. Metagenomic sequencing of sputum samples during antibiotic therapy revealed similar dynamics in clinical samples. This study shows that the complex microbial ecology dictates the outcomes of antibiotic therapy against a polymicrobial infection.

## Introduction

Antibiotics are our principal weapon against bacterial infection and one of the most widely prescribed medications worldwide. Global antibiotic consumption increased 39% from 2000 to 2015 and daily prescribed doses have increased by 65% [1]. Despite the broad use of these drugs, we still have a poor understanding of how they affect complex communities of microorganisms, as they are generally developed and tested against single pathogens in isolation. This is especially true in the case of the chronic lung disease cystic fibrosis (CF), where a polymicrobial infection develops early in life and evolves within the patient’s lung over decades [2]. Opportunistic pathogens, such as *Pseudomonas aeruginosa* and *Staphylococcus aureus*, tend to dominate these infections, but there is a more diverse microbial community contributing to lung disease in CF than originally appreciated [3, 4]. This complex community of microbes, termed the CF lung microbiome, includes not only opportunistic pathogens, the primary targets of antimicrobial therapy, but also other microorganisms originating from the upper airway, including anaerobic fermenters [5, 6]. Despite this complexity, antibiotic therapy against CF lung infections is frequently predicated on antimicrobial resistance profiles of isolates of classic pathogens from sputum samples. These bacteria are grown in pure culture, tested against a panel of antibiotics, and recommendations for treatment are suggested based on these in vitro antimicrobial susceptibility outcomes. It comes as no surprise, given the diversity of the CF lung microbiome now known [7, 8], that these susceptibility profiles are often unable to predict therapeutic outcomes [9, 10]. CF lung mucus is a complex microbial ecosystem, and we have a poor understanding of how antimicrobial therapies affect the collective community.

Understanding complex communities of organisms requires a comprehensive approach including in vivo and in vitro experiments supported by effective models. Experimental validation of these mathematical models are vital to their utility [11]. This approach has been successfully used in macroorganism ecology [12], where mathematical models of predator-prey relationships and community response to species extinctions have been used to help predict outcomes of these ecological disturbances [13]. In microbiology, however, these models are generally limited to single species populations and are difficult to apply to more complex systems [11, 14, 15]. Microbial communities represent most microbial lifestyles in the human body, making the need to better understand their structure and function dynamics of high importance. Recent efforts to model microbial systems have utilized flux-balance analysis based on bacterial metabolic networks and annotated genome reconstruction, also known as genome-scale metabolic models (GEMs) for the gut microbiota [16]. Stoichiometric models that predict the metabolism of specific nutrients from genomic information can demonstrate relevant cross-feeding and mutualistic interactions that can be validated in vitro [17]. Recent studies in CF have shown cross-feeding between communities of anaerobes and *Pseudomonas aeruginosa*, which is an important finding for translating metabolic models to clinical relevance [18]. Models that can predict experimental outcomes of perturbations in complex microbial communities are rarer and more challenging to develop, due to the difficulty in controlling community variability, in applying appropriate microcosms of the natural environment, and the generally poor understanding of how individual members interact in complex assemblages [19].

Our group recently developed a biofilm-based mathematical model that could predict CF lung microbial community shifts based on oxygen and pH gradients, setting a platform for experimental validation of the drivers of microbial dynamics in CF airways [20]. Here, we utilized this model of a simplified CF lung microbiome to predict outcomes of broad-spectrum and targeted antimicrobial therapy. We then explored in vitro results of paralleled experiments by exposing natural CF lung microbiomes to a panel of 11 different antibiotics in a culture microcosm called the WinCF system [21, 22]. Multi-omics experimental data was used to characterize outcomes of antibiotic therapy in the experiment and compared to model predictions. While some observed outcomes were expected, others were not, leading to further iterations of the model to better explain the complex effects of antibiotics on a polymicrobial community.

## Materials and methods

### Sample information and collection

Twenty-four sputum samples were collected from 24 CF volunteers in compliance with Spectrum Health (IRB project #2018-438), UC San Diego (IRB project #081500 and #160078), and the Health Insurance Portability and Accountability Act of 1996 (HIPAA) requirements. All samples were expectorated directly into a sterile sputum cup or 50 mL Falcon tube, immediately stored on ice, and shipped overnight on ice to the laboratory for antibiotic culture experiments and multi-omics analysis.

### WinCF culturing and inoculation

Artificial sputum medium (ASM) was prepared as described elsewhere [21, 22]. The pH was adjusted to 7.0 and 500 µL of ASM media was aliquoted into twelve 1.5 mL Eppendorf tubes per sputum sample (total *n* = 288). Each tube contained one of 11 different antibiotics mixed into the media prior to inoculation at the concentrations listed in Table S1 with one sample left as a control with no drug and an unioculated media control. Then 50 µL of diluted sputum from one of 24 subjects (5:1 in PBS) was used to inoculate each of the tubes and vortexed for 5 s. Triplicate glass capillary tubes were placed inside and allowed to fill with ASM via capillary action according to the method described by Comstock and colleagues [22]. These tubes were sealed at one end with Hemato-Seal capillary tube sealant (Fisher Biosciences) and placed into 15 mL tubes, plugged with a wet paper towel, and incubated at 37 °C horizontally over the course of 48 h. The remaining inoculated ASM was transferred to sterile 96-well deep-well plates (Thermo Scientific), covered, and incubated at 37 °C for 48 h.

### DNA isolation and 16S rRNA gene amplicon sequencing

DNA was extracted from 100 μL aliquots of media using a DNeasy PowerSoil HTP 96 kit (Qiagen) in 96-well plate format following the manufacturer’s protocol. An initial test PCR amplification was done on the V4 region with primers 27 F and 1492 R of the bacterial 16S rRNA gene to determine the efficacy of the DNA extraction. All PCR amplifications were performed in a thermocycler with the following program: 95 °C for 5 min for denaturation followed by 30 annealing cycles, 95 °C for 1 min, 48 °C for 30 s, 72 °C for 2 min, and finally, 72 °C for 10 min. PCR products were checked for amplification using agarose gel electrophoresis. For subsequent microbiome sequencing, the V4 hypervariable region of the 16S rRNA gene was amplified using Illumina compatible, dual indexed primers 515 f/806r [23]. PCR products were batch normalized using a SequalPrep DNA Normalization plate (Invitrogen) and product recovered from the plates was pooled. The pool was concentrated with an Amicon concentrator column (Millipore) and cleaned up using a 0.8x volume of AmpureXP magnetic beads (Beckman Coulter). The pool underwent quality control which included Qubit dsDNA HS, Agilent 4200 TapeStation HS DNA1000 and KAPA Library Quantification qPCR assays (Illumina). This pool was loaded onto one (1) MiSeq v2 Standard flow cell and sequencing was carried out in a 2 x 250 bp paired end format using a MiSeq v2 500 cycle reagent cartridge. Custom sequencing and index primers complementary to the 515 f/806r oligomers were added to appropriate wells of the reagent cartridge. Base calling was done by Real-Time Analysis (RTA, Illumina) v1.18.54. The output of RTA was demultiplexed and converted to FastQ format with Bcl2fastq v2.20.0 (Illumina).

### qPCR methods

Extracted DNA was amplified using the following primers 16S rRNA gene universal primers: 5′-TAC TAC GGG AGG CAG CAG-3′ (Forward) and 5′-GGA CTA CCA GGG TAT CTA ATC CTG TT-3′(Reverse) [24]. The reaction was performed in 12.5 µL using SYBR Green PCR master mix (Applied Biosystems). The cycle was run on a QuantStudio 7 in triplicate under the following conditions: 50 °C for 2 min, 95 °C for 2 mins for denaturation followed by 40 cycles, 95 °C for 15 s, 60 °C for 1 min. Standard curves of a diluted culture of *Pseudomonas aeruginosa* DNA with a known CFU/mL extracted with the same procedure were used to determine an estimate of the total rRNA gene copies per mL of media after adjusting for the four rRNA gene copies in the *P. aeruginosa* genome.

### Microbiome data processing

Raw sequences were processed using Qiita [25] and were quality filtered to generate amplified sequence variants using Deblur [26]. Sequences were aligned in QIIME2 version 1.9.1 [27] using MAFFT [28] to construct a phylogenetic tree using FastTree2 [29]. Taxonomy was assigned using the q2-feature-classifier against the 99% Greengenes 16S rRNA gene reference database (version 13-8) [30]. If the Greengenes database is updated this may result in identification of further diversity [30, 31]. QIIME2 version 2019.4.0 was used to calculate core diversity metrics, i.e. Shannon indices and weighted UniFrac distance matrices measures [27]. Amplicon sequence variants (ASVs) were classified as either pathogens or fermenters (anaerobes) based on their known clinical relevance in clinical microbiology labs and CF literature [4, 32] (Table S1 and S2).

### Metabolomics

Organic extraction was performed by adding twice the sample volume (400 μl) of chilled 100% methanol, vortexing briefly, and incubating at room temperature for 2 h. Samples were then centrifuged at 10,000 x g for 10 min and the supernatant was collected [33] Methanolic extracts were analyzed on a Q-Exactive Hybrid Quadrupole-Orbitrap mass spectrometer coupled to a Vanquish ultra-high-performance liquid chromatography system (Thermo) in positive ion mode. Briefly, sputum metabolites were separated on a Aquity C18-Reverse phase column (Waters) with a 12 min chromatography run using an acetonitrile and water gradient (98:2 to 2:98). The injection volume was 10 µL, the flow rate was 0.40 mL min^−1^, and the column temperature 60 °C. Full MS^1^ survey scans and MS^2^ mass spectra for five precursor ions per survey scan were collected using electrospray ionization with a scan range set from *m/z* 100 to 1500 for the full MS mode (minutes 1–10 of run) [4, 33]. All raw files were converted to.mzXML format and then processed with MZmine 2.53 software [34], GNPS molecular networking [35], and SIRIUS [36].

### Modelling methodology

A mathematical model, initially developed elsewhere [20], was employed, consisting of two microbial entities, one denoted as the pathogen (representing primarily, but not exclusively, *Pseudomonas aeruginosa*) and the other as a representative anaerobic fermenter, cohabiting a columnar domain of height 0.8 cm, together with a number of chemical species (oxygen, nitrogen, sugar, amino acids, ammonium, acid, inhibitor, and antibiotics) which diffuse through the domain and may be consumed or produced locally by the microbes, and which may locally influence microbial growth and death rates. The model consists of ten reaction-diffusion partial differential equations (for diffusible chemical species) coupled with two spatially dependent ordinary differential equations (for microbial entities), and equations are solved numerically (see supplemental Material for details). Parameters of the model are based on previous experiments with these two communities in WinCF and knowledge from the literature (Table S2) [20]. The model dictates that fermenters consume sugar and produce acid, while the pathogen consumes oxygen, nitrate, and amino acids. The pathogen produces ammonium from deamination and a fermenter inhibitor, meant to represent its antibiotic production from compounds such as phenazines and rhamnolipids [21, 37]. Fermenter growth rate decreases with increasing oxygen concentration, and the fermenter carrying capacity decreases with increasing concentration of inhibitor. The carrying capacity of sputum for the pathogen is an increasing function of pH (or equivalently a decreasing function of the acid), which is based on our initial study that showed the bacterium is easily outcompeted at lower pH [20]. Three different antibiotic types are deployed: one which only kills fermenters (denoted T_f_), one which only kills *P. aeruginosa* (denoted T_p_), and one which kills both (denoted T_w_). We suppose that a fixed amount of antibiotics are added at time *t* = 0 with uniform concentration in the reactors, then consumed while killing bacteria, and that the diffusion coefficients, killing rates, and consumption rates are the same for all antibiotics.

### Metagenomic sequencing of patient sputum samples

DNA from sputum samples was isolated using the Dneasy PowerSoil Pro Kit (Qiagen), according to the manufacturer’s protocol. Isolated DNA was quantified by Qubit. Metagenomics was performed by CosmosID Inc. (Rockville, MD) according to their standard protocols. DNA libraries were prepared using the Nextera XT library preparation kit (Illumina), with a modified protocol. Library quantity was assessed with Qubit (ThermoFisher). Libraries were then sequenced on an HiSeq platform 2x150bp (Illumina). Unassembled sequencing reads were directly analyzed by CosmosID metagenomic software (CosmosID Inc., Rockville, MD) via high-performance data-mining k-mer algorithm and highly curated dynamic comparator databases that disambiguate short reads. Taxonomy was assigned via the CosmosID database. Diversity measures (Shannon index) were calculated via the CosmosID metagenomic software. Identified sequences were classified as either pathogens or fermenters based on their clinical relevance (Table S2).

### Follow-up validation experiments with representative T_f_, T_p_, and T_w_ antibiotics

ASM was supplemented with 0.04 mg/L of phenol red pH indicator and subsequently adjusted to 7.4. Three antibiotics (meropenem trihydrate (T_w_), tobramycin (T_p_), and metronidazole (T_f_)) were chosen and added as individual treatments at a concentration of 2.05 mg L^−1^. These were then incubated with communities obtained from sputum preserved in 50% glycerol, one community was from a patient known to have a *P. aeruginosa* infection and the other was not known to have *P. aeruginosa* according to clinical records. This was verified by plating the sputum culture on cetrimide agar for *P. aeruginosa* growth. Then, 400 µL of inoculated ASM media was aliquoted into 1.5 mL Eppendorf tubes in replicates of 10 and incubated for 48 h at 37 °C. DNA was extracted from 50 µL of the sample via Quick-DNA Miniprep Plus kit (Zymo Research Corp) followed by qPCR and 16S rRNA gene sequencing as described above. This experiment was repeated with 5 replicates for validation. The pH estimation was obtained by measuring the average RGB color values from the phenol red media dye using Image J software and comparing it to ASM with phenol red standard buffered from pH 5–8.

### Statistical analysis

Alpha diversity for microbiome and metabolome were calculated with the Shannon diversity index. Beta diversity for the microbiome was calculated using the weighted UniFrac distance while Bray-Curtis was used for the metabolome. Permutational Multivariate Analysis of Variance test (PERMANOVA) individual and mixed effect model, examining interactions between and nested effects, were calculated from the relative abundances via the Bray-Curtis method with 999 permutations. Subsequent stepwise model selection was used to determine which effects/mixed effects had the greatest influence on ASV presence/absence. Kruskal-Wallis tests were done to determine significance across various measures as the data was not assumed to be normal. Post hoc Mann-Whitney tests with applied Bonferronic correction (Tables S4–S13) were performed to examine differences across the antibiotic treatments. Prevalence measures on ASVs were also examined (Fig. S9). PERMANOVA and the standard homogeneity condition was performed with the R vegan package (v.2.5–7) [38] via adonis() and betadispr(). Kruskal-Wallis tests and post-hoc Mann-Whitney tests were performed using the R dplyr package (v.1.0.2) [39]. Data visualization was done using the R package ggplot2 (v.3.3.5) [40] and phyloseq (v.1.30.0) [41]. A PCA was calculated via the R package stats (v.3.6.2) and visualized via R package factoextra (v.1.0.7). Prevalence was measured and visualized using the R package microbiome (v.1.17.41) [42].

## Results

### Model overview and parameters

**Fig. 1:**
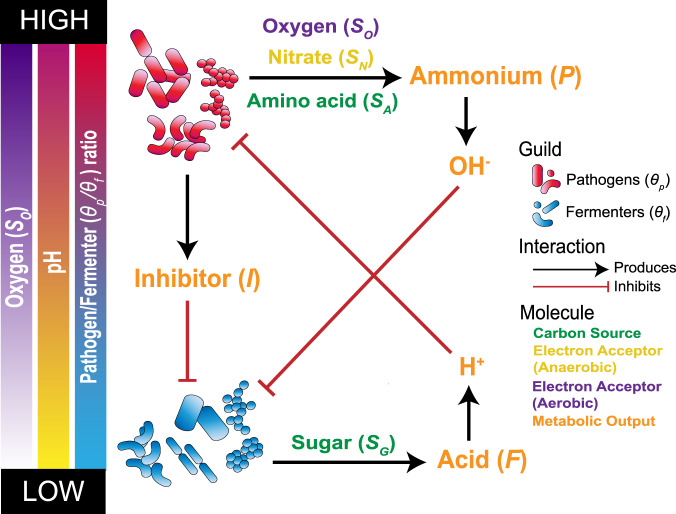
Schematic of principles and interacations defining the mathematical model. All consitunents of the model are represented in illustrating basic assumptions and interactions. Fermenters (*θ*_*f*_) metabolize (*S*_*G*_) as a carbon source, which produce acid (*F*) leading to an increase in hydrons (H^+^) (i.e. lowering the pH) under anaerobic conditions. This pH decrease inhibitis the growth of pathogens. Pathogens (*θ*_*P*_) in the presence of oxygen (*S*_*O*_) (i.e., aerobic conditions) use amino acids (*S*_*A*_) as their primary carbon source. The byproduct of this metabolism is ammonium (*P*), which produces hydroxide (OH^-^) leading to an increase in pH, inhibiting fermenter growth. Under anaerobic conditions pathogens use nitrate (*S*_*N*_) as an electron acceptor. In addition to this pathogens produce a chemical inhibitor of fermenters (*I*).

Our mathematical model of the CF lung microbiome dynamics, originally developed in [20], is based on knowledge of the physiology and interactions among community members from experimental data and evidence in the literature. The model setting is a mucus-plugged tube, open to the air at the top and sealed at the bottom, mimicking a lung bronchiole. This setting is meant to pair with a previously established experimental microcosm called the WinCF system [21], which we use below for experiments. There is an important spatial component to the model, as oxygen penetration from the open top of the tube is constant and shapes the community structure. The consequences of these chemical gradients were first modelled in our initial study [20]. The community members are classified as either “pathogens”, representing classic CF pathogens, or “fermenters”, representing other anaerobic organisms commonly encountered in CF airways. These classifications are a significant simplification, but they can be considered as guilds, in that their individual members have similar inherent properties defined by their core metabolism, antibiotic resistance, and niche occupancy [20]. The definition of classic pathogens and anaerobic fermenters is also clinically relevant, as the former are those assayed in clinical labs for antibiotic resistance to inform treatment decisions, whereas anaerobic fermenters are not cultured or tested for susceptibility in most clinical labs. Classifications of each microbiome member into these guilds are available in Tables S2–S4. Fermenters reside in low oxygen areas and utilize sugars to produce acids [20] (Fig. 1). Pathogens, principally, but not exclusively, *Pseudomonas aeruginosa*, occupy high oxygen regions where they aerobically respire and utilize amino acids as a carbon source producing ammonium, which increases the surrounding pH [20] (Fig. 1). Pathogens can also respire anaerobically, with nitrate as an electron acceptor (Fig. 1). In addition to increasing the surrounding pH, they produce inhibitor molecules (such as phenazines and quinolones) that inhibit the growth of fermenters [20] (Fig. 1). This model is hereon referred to as the “*mathematical model”*.

### Predicting and modelling outcomes of antibiotic therapy

To better conceputalize and compare our modeling and experimental results, we first theoretically predicted the outcomes of antimicrobial therapy against the two guilds using three theoretical drugs: one with fermenter coverage (denoted T_f_), one with pathogen coverage (denoted T_p_), and one with broad spectrum coverage (denoted T_w_). This approach is hereon referred to as the “*theoretical prediction*”. To further enable comparison to experimental data we outline characteristics of the two guilds we expect to observe in the experiments. Firstly, the growth of anaerobic fermenters is positively correlated with an increase in gas production (bubble formation in the WinCF system) [21]. Second, an increase in *P. aeruginosa* positively correlates with an increase in its inhibitor molecule (e.g., Quinolone HHQ) and *P. aeruginosa* does not produce gas in the WinCF system [21]. Thirdly, based on Tables S1–S4 and the CF microbiome literature, fermenters are more diverse than pathogens [2, 43, 44]. These characteristics of our *theoretical* *prediction* enable direct comparison to microbiome measures of experimental results, such as alpha diversity, beta diversity, pathogen relative abundance, fermenter relative abundance and total bacterial load (TBL).

**Fig. 2:**
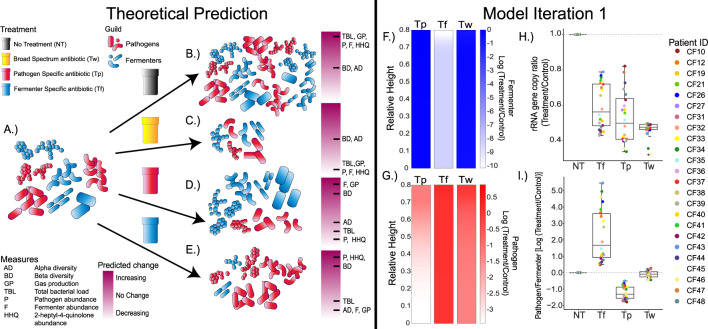
Theoretical predictions and Model iteration 1. The initial microbiome is composed of both pathogens and fermenters and is illustrated in (**A**), but the proportions of these are unique to each patient. Under pressure of the various treatments (**B**) NT, (**C**) T_w_, (**D**) T_p_, and (**E**) T_f_ the predicted community response is illustrated. The response i.e., (expected change) in common microbiome measures as indicated in the legend (yellow = increase, red = decrease). The measures are the following: Alpha diversity (AD), Beta diversity (BD), gas production (GP), total bacterial load (TBL), pathogen abundance (P), fermenter abundance (F), and 2-heptyl-4quinolone abundance (HHQ). The model output treatment-to-NT log-ratio of (**F**) fermenter population and (**G**) pathogen population of patient 12 as an example with spatial variation at *t* = 50 h. Boxplots showing model outcomes of the (**H**) 16S rRNA gene copy ratio and (**I**) Pathogen to Fermenter log-ratio compared to the control. Each patients’ actual sputum Pathogen/Fermenter ratio was used as input to the model (*n* = 24). The dotted grey line denotes no change from treatment.

With our theoretical prediction we expect the following outcomes when communities are exposed to antibiotics: (1) community resistance, (2) community death, (3) pathogen death, and (4) fermenter death (Fig. 2A–E). In both the complete absence of an antibiotic and community resistance, we expect TBL, pathogens, fermenters, HHQ, and gas production measures to increase until reaching carrying capacity (Fig. 2B). The opposite, community death (treatment with T_w_) results in both microbial entities failing to grow (Fig. 2C). T_w_ treatment would not change alpha or beta diversity, as we would simply measure the initial inoculum due to total community death. Outcomes 1 and 2 have a degree of uncertainty due to the fact that it is difficult to assume the community would not change from the inoculum without an antibiotic present, but it is expected that T_w_ would have less impact on microbiome diversity than T_p_ or T_f_ (Fig. 1C). Treatment with T_p_ results in an anaerobic fermenter bloom, increasing alpha and beta diversity along with gas production and a decrease in HHQ production (Fig. 2D). Finally, in the case of T_f_ treatment, fermenter abundance and gas production would decrease while HHQ abundance would increase (Fig. 1E). Treatment with T_f_ will also result in a decrease in alpha diversity and an increase in beta diversity because of changes in community structure when the diverse anaerobic fermenters are killed (Fig. 2E).

The *theoretical prediction* was then tested with the *mathematical model* hereon referred to as “*model iteration* 1”. Importantly, our model parameters can use relative abundance data of the two guilds as input. Therefore, we used the sputum microbiome data of all 24 subjects as inputs for *model interation 1* (Fig. 2F–H). The outputs were in line with our *theoretical prediction* and showed that the fermenter drug would reduce the fermenter load, with little effect on the pathogens, the pathogen drug vice versa, and the broad-spectrum antibiotic would kill both (Fig. 2F–H). However, *model iteration 1* did produce some unexpected results. The TBL of the T_w_ decreased to similar levels as T_f_ and T_p_, indicating similar levels of killing whether there was selection against a single guild or the whole community (Fig. 2H). In addition, the TBL and Pathogen/Fermenter log-ratio were variable, indicating the carrying capacity and community dynamics were predicated upon characteristics of this initial sputum inoculum (Fig. 2F–H). Our *theoretical prediction* (Fig. 2A–E), in tandem with *model iteration 1* (Fig. 2F–H), provided a platform for comparison to the in vitro antibiotic experiments with the WinCF system described below.

### Experimental results of antibiotic therapy against the lung microbiome

We examined the effects of antibiotics (*n* = 11) on the CF sputum microbiome cultured in a lung bronchiole microcosm (WinCF system, *n* = 24) using a combination of 16S rRNA gene amplicon sequencing, metabolomics, and qPCR analysis and compared to our *theoretical prediction* and *model iteration 1*. This is hereon referred to as the “*antibiotic experiment*”. The antibiotics were chosen to represent the main chemical classes commonly used in CF clinics and included: amoxicillin, azithromycin, aztreonam, ciprofloxacin, colistin, doxycycline, levofloxacin, meropenem, metronidazole, bactrim (a combination of sulfamethoxazole/trimethoprim), and tobramycin. Each of the 24 sputum samples were used as an incoculum in ASM treated with one of 11 different antibiotics cultured at 37 °C for 48 h (Table S1) and compared to a no-treatment control. WinCF tubes were also inoculated with this media/sputum/antibiotic mixture to quantify gas bubble production from fermentation (as described in [21]). The antibiotic concentration for each drug was variable and chosen to match the measured concentrations in the blood or sputum of pwCF in pharmacokinetic studies (Table S1). The most prominent genera across all samples after growth were *Pseudomonas, Streptococcus, Veillonella, Haemophilus, Fusobacterium, Prevotella, Staphylococcus, Achromobacter*, and *Neisseria* (Fig. S2). A principal component analysis (PCA) biplot, examining the top five factors by percent contribution, showed the primary genera driving community differentiation were *Pseudomonas, Streptococcus*, and *Staphylococcus* (Fig. S3). The effects of antibiotics and individual patients on the composition of the communities were compared via PERMANOVA (Table S7). Tested separately, both antibiotic and subject source had a highly significant effect on the community structure (*p* < 0.001). However, the nested effect and interactions between antibiotic and the patient did not (*p* = 1). Thus, the changes in the ASV composition were the result of both the antibiotic and the subject’s initial community separately, but there were not universal responses across subjects for each drug.

**Fig. 3:**
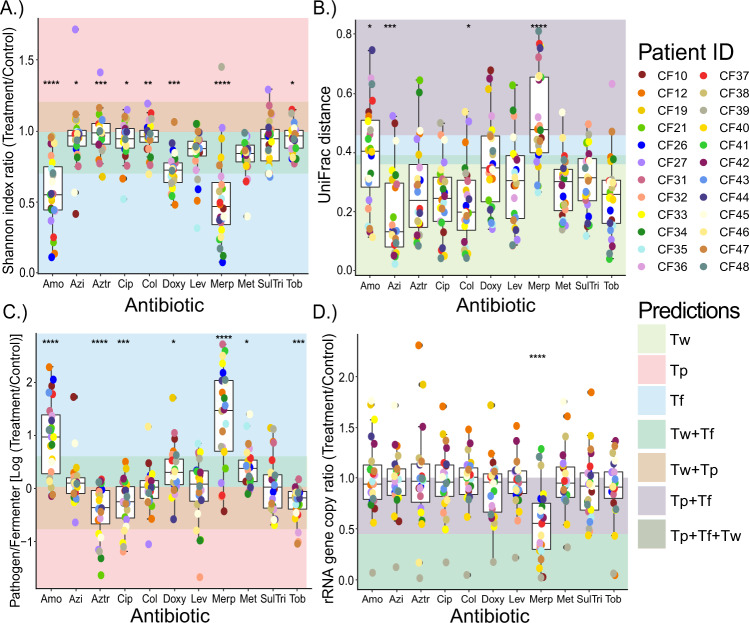
Different microbiome community measure changes compared to the no-antibiotic control. The impacts of antibiotics (*n* = 11, Amo = amoxicillin, Azi = azithromycin, Aztr = aztreonam, Cip = ciprofloxacin, Col = colistin, Doxy = doxycycline, Lev = levofloxacin, Merp = meropenem, Met = metronidazole, SulTri = bactrim and Tob = tobramycin) compared to untreated control samples on (**A**) Shannon index ratio, (**B**) Weighted UniFrac distance, (**C**) pathogen to fermenter log ratio, and (**D**) rRNA gene copy ratio. Individual points are colored by patient (*n* = 24). The shaded areas behind the boxplots are regions of the plot where the outcomes of our theoretical predictions and/or model iteration 1 would lie if correct, colored according to antibiotic treatment type (T_w_, T_p_, and T_f_). Kruskal-Wallis statistics are reported in Table S8. Asterisks denote *p*-value significance where ****p ≥ 0.0001, ***p ≥ 0.001, **p ≥ 0.01, *p ≥ 0.05. Mann-Whitney post hoc tests are reported in the Supplementary material (Tables S10–S16).

We visualized the changes in our microbiome and physiology measures compared to the no antibiotic control in the context of the *theoretical prediction* (colored areas in Fig. 3) to aid the identification of outcomes that did or did not match the predictions. All measures had significantly different changes across antibiotics according to a Kruskal-Wallis test, except for HHQ abundance (Tables S8, S9). Alpha diversity (Shannon index) showed a general decrease compared to an untreated control when the antibiotic was applied (Fig. 3A), but this depended on the antibiotic. amoxicillin and meropenem resulted in the strongest decreases in alpha diversity, being significantly lower than the other treatments (Table S10), which changed little and had instances of increases in diversity (Fig. 3A and Table S10). Beta diversity (weighted UniFrac distance) comparisons of treatment samples to the no antibiotic control enabled quantification of the degree of microbiome change due to treatment. amoxicillin and meropenem had the highest beta diversity increases, with the latter being significantly higher than 8 others (Table S11) and azithromycin the lowest (Tables S9, S11), though there was significant variability within each drug limiting the statistical significance across the different treatments. The variability in the *antibiotic experiment* showed that although some drugs had smaller impacts than others all antibiotics impacted the microbiome composition with some unique responses for particular patients (Figs. 3B and S4). Plotting Pathogen/Fermenter log-ratio changes compared to the control enabled the quantification of dynamics between the two guilds and direct comparisons to the *theoretical prediction* and *model iteration 1*. Again, amoxicillin (significantly higher than 7 of 10) and meropenem (higher than 8 of 10) increased the relative abundance of pathogens compared to fermenters. Significant decreases in this ratio were observed with aztreonam, tobramycin, and ciprofloxacin (Fig. 3C and Tables S8 and S12). An unexpected result not identified by *theoretical predictions* or *model iteration 1* was observed when comparing TBL changes between treatment samples and controls. Overall, the rRNA gene copy number (a measure of total bacterial abundance using qPCR) did not change significantly across the different antibiotics, except for meropenem, which significantly reduced this ratio compared to 8 of 10 treatments (Fig. 3D, Tables S8 and S13). Interestingly, despite the decrease in alpha diversity and increase in beta diversity compared to the control, amoxicillin did not have a significant decrease in TBL. Furthermore, all drugs had samples that increased in total bacterial abundance (i.e. values above 1 in Fig. 3D). Specifically, 17.8% of all samples showed a 20% increase in rRNA gene copies and 6.8% increased by 40% (Fig. 3D and Tables S17, S18). Therefore, despite the presence of an antibiotic meant to inhibit bacterial growth, the total carrying capacity increased in many samples of the *antibiotic experiment*, but this phenomenon was not driven by a specific drug. HHQ abundance changed dynamically with antibiotic treatment (greater than 2-logs) and these changes were mostly driven by the individual subject source not a specific antibiotic (Tables S14, S15 and Figs. S4a, S4e), meaning that there was a more personalized response to the production of this *P. aeruginosa* associated-metabolite. Finally, gas production, our measure of microbial fermentation in the WinCF system, showed an overall decreasing trend compared to the control, most pronounced from meropenem, doxycycline and amoxicillin, but few comparisons were significant due to extensive variation within each treatment (Fig. S3a and Table S16). Similarly to the increases in TBL, but this time predicted by the model, increases in the gas production were seen in the experiment and all antibiotics had at least one instance of an increase compared to the no-treatment control (Fig. S4a and Table S16).

### Characterizing outcomes of antibiotic therapy against the CF lung microbiome

**Fig. 4:**
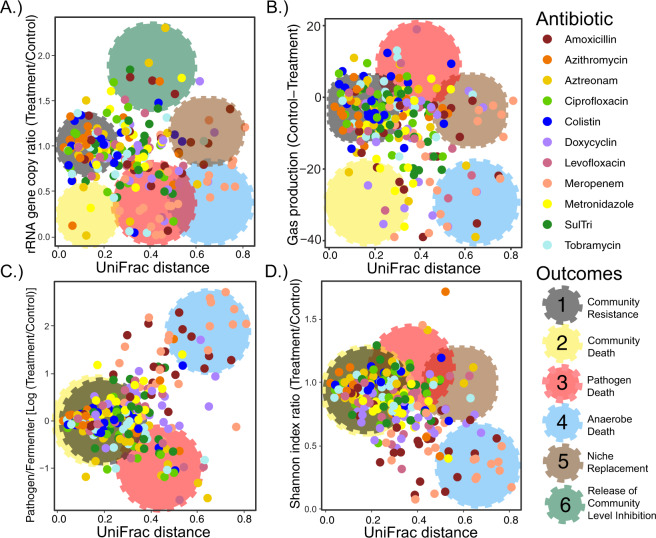
Characterizing outcomes in the *antibiotic experiment*. Weighted UniFrac distance compared to (**A**) rRNA gene copies, (**B**) Gas production, (**C**) Pathogen to fermenter log ratio, (**D**) Shannon index. Individual points are colored by antibiotic treatment (*n* = 11). Observed outcomes (Community resistance, community death, pathogen death, anaerobe death, niche replacement, and release of community level inhibition) are highlighted via large cogs on each of the panels colored by the outcome they represent. These highlighted regions are meant to aid in visualization of their presence in the overlying data. Cutoff values of for the outcomes are further described in Table S17.

To better quantify and characterize outcomes from the *antibiotic experiment*, microbiome measures of interest were plotted against the UniFrac distance from the control sample (Fig. 4). Four outcomes observed from this experiment matched the *theoretical predictions* and *model iteration 1* including: 1) community resistance, 2) community death, 3) pathogen death, and 4) anaerobe death (outcome definitions quantified in Table S17). Outcomes five and six were not predicted and were defined as 5) *niche replacement* events and the 6) *release of community level inhibition*. The most common outcome was 1) community resistance, which encompassed 44.6% of all samples tested (Fig. 4, quantified outcome definitions available in Tables S17, S18). This may indicate that the CF lung microbiome has an inherent antibiotic resistance due to decades of exposure and the propensity of its constituents to grow as biofilms [45, 46]. Community death (outcome 2), occurred 17.8% of the time. Cases of community death with little change in beta diversity were rare, indicating that comprehensive antibiotic killing most often results in a community structure change compared to a no antibiotic control. Both pathogen death (8%) and fermenter death (17%) outcomes were observed in our experiments (Fig. 4 and Tables S17, S18). Anaerobe death outcome was driven by meropenem and amoxicillin as shown in Fig. 3C, whereas, pathogen death was not driven by any particular drug. Niche replacement (outcome 5) occurred when the TBL of the sample did not change but the UniFrac distance was above 0.4, which encompassed 6.4% of samples (Fig. 4b, d). This outcome may reflect the diverse nature of the fermenter guild; when a certain species is killed, another can take its place, maintaining the fermentative nature of the community but resulting in a community structural change. The *release of community level inhibition* (outcome 6) was defined as an increase in TBL (>40%), which occurred in 6.8% of samples. The microbiomes of outcome 6 were predominantly dominated by pathogens compared to the control samples (Fig. S7). We found this outcome to be especially interesting, with potential clinical relevance; we therefore performed follow up experiments to understand it further.

Other interesting data relationships were found in these experiments (Fig. S8) though they were not defined as outcomes. For example, the changing UniFrac distance and change in alpha diversity were negatively correlated (Fig. S8a). A large increase in UniFrac distance (over 40% increase), was generally associated with takeover by a particular ASV, driving this phenomenon (Figs. S7 and S9). According to prevalence measures of theses samples the prominent genera in these instances were *Pseudomonas* and *Streptococcus* (Fig. S9). In the cases of meropenem and amoxicillin, UniFrac distances were increased while the Shannon indices were decreased, due to the killing of diverse anaerobic community, but there were fewer cases of an increase in alpha diversity and a significant microbiome change (observed in 3 samples only) indicating a kind of buffering of the microbiome by the diverse anaerobic community (Fig. S7a). The increase in TBL characterizing outcome 6 was rarely associated with an increase in alpha diversity (Table S17). Finally, similar to a phenomenon described in CF sputum [31], when the microbiome alpha diversity increases the metabolome diversity decreases, likely reflecting consumption of different metabolites by a more diverse microbiome (Fig. S7c).

### Model iteration 2 and experimental validation to explain increase in TBL

**Fig. 5:**
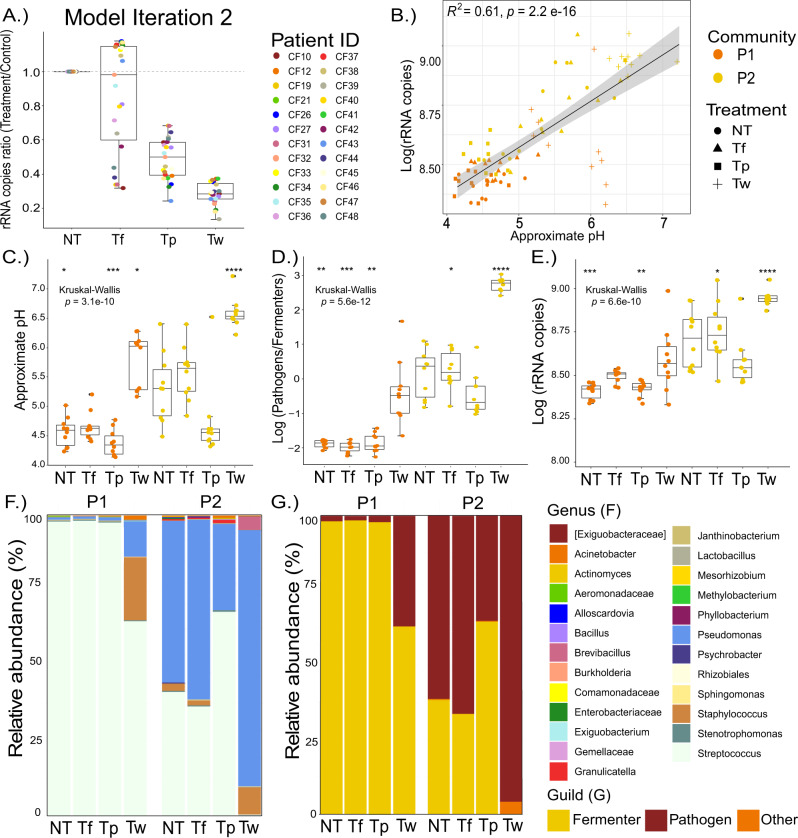
Model alteration and verification. (**A**) Model iteration 2 outcomes of 16S rRNA gene copy ratio of each patients’ actual sputum Pathogen/Fermenter ratio was used as input to the model (*n* = 24). Individual points are colored by antibiotic treatment (*n* = 11). The dotted grey line denotes no change from treatment. Subsequent experimental validation using two communities, P1 and P2 (*n* = 10), showing the (**B**) pH in relation to log rRNA gene copies, (**C**) Approximate pH, (**D**) Pathogen/Fermenter log ratio, (**E**) log rRNA gene copies, (**F**) Genera abundance, (**G**) Distribution based on genera-classification as classical pathogen or anaerobic fermenter. Asterisks denote *p-*value significance where ****p ≥ 0.0001, ***p ≥ 0.001, **p ≥ 0.01, *p ≥ 0.05.

Because *model iteration 1* did not predict the interesting outcome 6, we altered its parameters to determine if we could observe an increase in TBL in the presence of an antibiotic, hereon referred to as “*model iteration 2*”. In *model iteration 1*, parameter λ in the function g2(Z) was set to 0.1, which represents pH driven inhibition of fermenters on pathogen growth. Due to the inverse relationship of this parameter, reducing it to 0.05 increased the strength of inhibition, resulting in an increase in TBL for some subjects, akin to that observed in our experimental outcome 6 (Fig. 5A). This only occurred in T_f_ treatments in *model iteration 2*, corresponding to a bloom in pathogens after killing of anaerobes. Furthermore, this phenomenon was only present in modelled samples that initially contained much lower populations of the fermenter guild compared to pathogens and is dependent on the spatial structure driven by oxygen gradients that is an inherent property the modeled system (Figs. 1 and 5A). This finding suggests that outcome 6 in the *antibiotic experiment* may be driven by an antibiotic mediated release of community level inhibition driven by the effect of low pH from fermenters on pathogens and the inhibition of anaerobes by oxygen [20]. Thus, we set out to explore this phenomenon in more detail experimentally.

A simple in vitro experiment was performed where three antibiotics, meropenem (T_w_), tobramycin (T_p_), and metronidazole (T_f_), were added at 2.048 mg/L in ASM media inoculated with two representative communities obtained from pwCF: P1 and P2 (*n* = 10 replicates) (Fig. 5B–F). The three drugs were selected based on their common uses against CF infections based on pathogen and/or anaerobic coverage, but we acknowledge that their effects are not exclusive to these organisms. Community P1 did not contain *P. aeruginosa* via culturing on cetrimide agar, whereas the bacterium was isolated from the sputum of P2. This provided a unique opportunity to test the predictions from *model iteration 2* on the outcomes of a community with or without *P. aeruginosa*. A lower concentration of antibiotics was chosen to avoid widespread killing of the communities. We examined the following: rRNA gene copies, approximate pH (based on RGB color values inferred from phenol red buffered media standards) and 16S rRNA gene amplicon sequencing (Fig. 5). This is hereon referred to as the *validation experiment*. The *validation experiment* reproduced outcome 6, where both the number of rRNA gene copies were higher when the antibiotic was present than in the no treatment control for both P1 and P2 (Fig. 5C). In contrast to *model iteration 2*, this only occurred in treatment T_w_ (paired t-test, *p* = 0.000831) (Fig. 5). Accordingly, this increase in TBL corresponded to an increase in pH of the cultures, validating the association of the anaerobe induced fermentation with an inhibition of the communities’ total carrying capacity (*p* = 1.69 × 10^−9^, Fig. 5B–E). In fact, there was a strong positive correlation between the TBL and media pH overall (Fig. 5B). Furthermore, P2 reached a higher bacterial load overall than P1 in the *validation experiment*, indicating that the pathogen’s presence drove the community to a higher carrying capacity (Fig. 5E). The lower growth in community P1 shows that a community of primarily anaerobic fermenters struggles without the aerobic pathogen present. Microbiome profiles of these follow up experiments validated the predictions of *model iteration 2* and initial findings of outcome 6 (Fig. 5F, G). Meropenem killed the anaerobic community (primarily *Streptococci*) and the increase in TBL was driven by a bloom of *Pseudomonas* (P2 community) and *Staphylococcus* (P1 community) to a higher level than the communities’ inherent carrying capacity (Fig. 5F, G). This experiment was subsequently repeated (*n* = 5), with the same results observed (Fig. S10). It was interesting that a similar increase in TBL occurred from a community without a dominant pathogen (P1, Fig. 5G). We hypothesize that this result is due to the importance of both oxygen and pH in the governing dynamics. With very low levels of the pathogen guild, the community struggles to grow due to high oxygen penetration. When the anaerobes are inhibited by antibiotics, even low levels of an initial pathogen can begin to bloom, as they are not inhibited by oxygen or the antibiotic, and this leads to an increase in total carrying capacity.

### Antibiotic effects at the strain level in pwCF

**Fig. 6:**
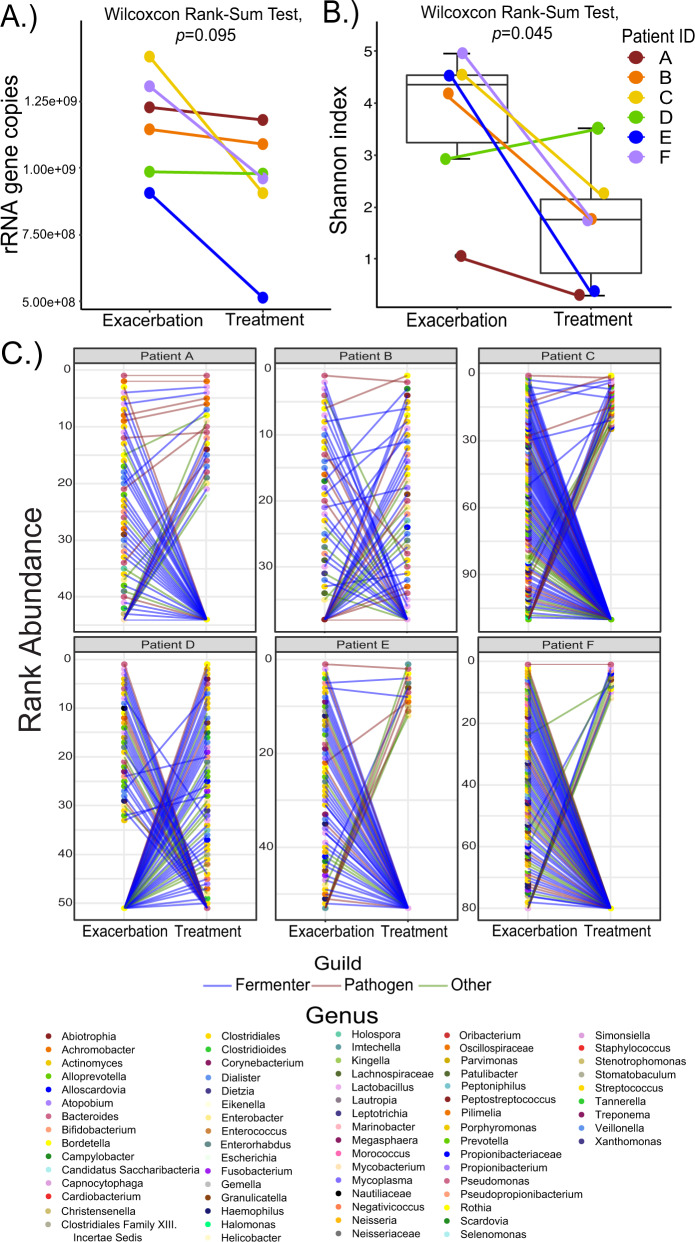
In vivo changes across individuals. qPCR and shotgun metagenomics were performed on sputum samples from individuals (*n* = 6) before and after exacerbation. We examined the following: (**A**) rRNA gene copies (**B**) Shannon Index, and (**C**) Rank abundance. Each point on the rank abundance represents an individual strain. The color of lines on the rank abundance represents type of bacterium based on our model definitions where blue equates to Fermenters, red to Pathogens, and green to other.

To explore similar phenomena in outcomes 5 and 6 from pwCF treated with antibiotics we sequenced the metagenomes of sputum samples collected from subjects immediately prior to and during antibiotic treatment (*n* = 6) (Table S19). To minimize the effects of multiple therapies at once, a common occurrence in CF therapeutics, these samples were selected based on the treatment provided being the only known antibiotic prescribed to the subject at the time. Metagenomes were analyzed at the strain level and TBL was examined using qPCR. Overall, there was no significant decrease in TBL (Fig. 6A, Wilcoxon rank-sum test, *p* = 0.095), but alpha diversity significantly decreased (Fig. 6B, Wilcoxon rank-sum test, *p* = 0.045). Analysis of the rank abundance changes of the microbiome at the strain level showed that all six subjects had dynamic changes in their sputum microbiomes associated with antibiotic treatment despite little decrease in TBL (Fig. 6C). Thus, like outcome 5, and indicative of outcome 6, dynamic community changes occur in pwCF with minor changes in TBL.

## Discussion

Outcomes of antibiotic therapy against pulmonary infections in cystic fibrosis and other chronic lung diseases have poor predictability. Recovery to baseline from a pulmonary exacerbation after therapy occurs in 75% of patients treated, thus, one quarter of these subjects do not return to their previous baseline lung function [47]. The choice of antibiotic treatment is generally predicated on antibiotic resistance profiles determined from isolates of lung pathogens, but is poorly informative of subsequent clinical outcomes [48]. This is not surprising, as this approach is naïve to the fact that lung infections represent a polymicrobial ecosystem with interdependencies and competition among its members [31, 49]. Here, we aimed to first predict with modelling, and then explore in detail with experimentation, the outcomes of antibiotic therapy against a cultured CF lung microbiome. Four of our six outcomes were predicted theoretically and by modelling, but two were not, leading to further exploration of their origins. Our experiments indicate that the CF lung microbiome may have an inherent resistance to antimicrobial therapy, as many antibiotics had little overall effect on the communities, but meropenem and amoxicillin had dynamic impacts, particularly through killing of anaerobic fermenters. In some instances, regardless of antibiotic chosen, the community reached a higher carrying capacity when the drug was present than when antibiotic free. This finding exemplifies the inherent ecology within this microbial system that we define as *community level inhibition*. The *mathematical model iteration 2* was able to provide clues as to why this occured, demonstrating the utility of the modeling approach. Fermentation by anaerobic bacteria in the community produces acid that is known to inhibit pathogens, particularly *P. aeruginosa* [20]. Killing of these fermenters raises culture pH allowing a bloom of the pathogen to reach a higher carrying capacity than the community without an antibiotic.

We previously showed that pH shapes the niche space of the CF microbiome [20] and have now shown that low pH also limits the total carrying capacity of the system. Our experiments also show that the presence of an aerobic pathogen enables the community to reach a higher overall load, likely due to oxygen draw down from aerobic metabolism creating the anaerobic niche needed for fermenters. There is evidence that similar dynamics may occur in pwCF during treatment for pulmonary exacerbations [4, 21]. Thus, further study on the temporal assembly of the CF lung mcirobiome is needed, which can also be supported by modeling. These complex ecological consequences of disturbance may help explain why treatment outcomes have been so difficult to predict for CF. All microbiomes in humans and animals contain complex species-species and guild-guild interdependencies and competitive interactions. It is paramount that we understand these interactions to enable better therapeutic outcomes of antibiotic therapy against any microbiome, pathogenic or commensal.

## Supplementary information

Supplementary information

Supplementary tables

## Data Availability

The 16S rRNA gene amplicon microbiome data was deposited in the Qiita [25] database as the project numbers 12992 and 14086. The metagenomic data was deposited to the NCBI database under the SubmissionID: SUB11489970 and BioProjectID: PRJNA839435. The code for simulating the mathematical model is available at https://github.com/zhangtianyu-msu/WinCF_Antibiotic_Code.
