# Supplementary material for: Complex and unexpected outcomes of antibiotic therapy against a polymicrobial infection
Source: ISME J. 2022 May 21;16(9):2065–75. doi: 10.1038/s41396-022-01252-5 (PMC9381758; doi:10.1038/s41396-022-01252-5)
Supplement: Supplementary file 1 — Supplementary information [file 41396_2022_1252_MOESM1_ESM.docx]

**Supplementary Material**

# Model Equations

**
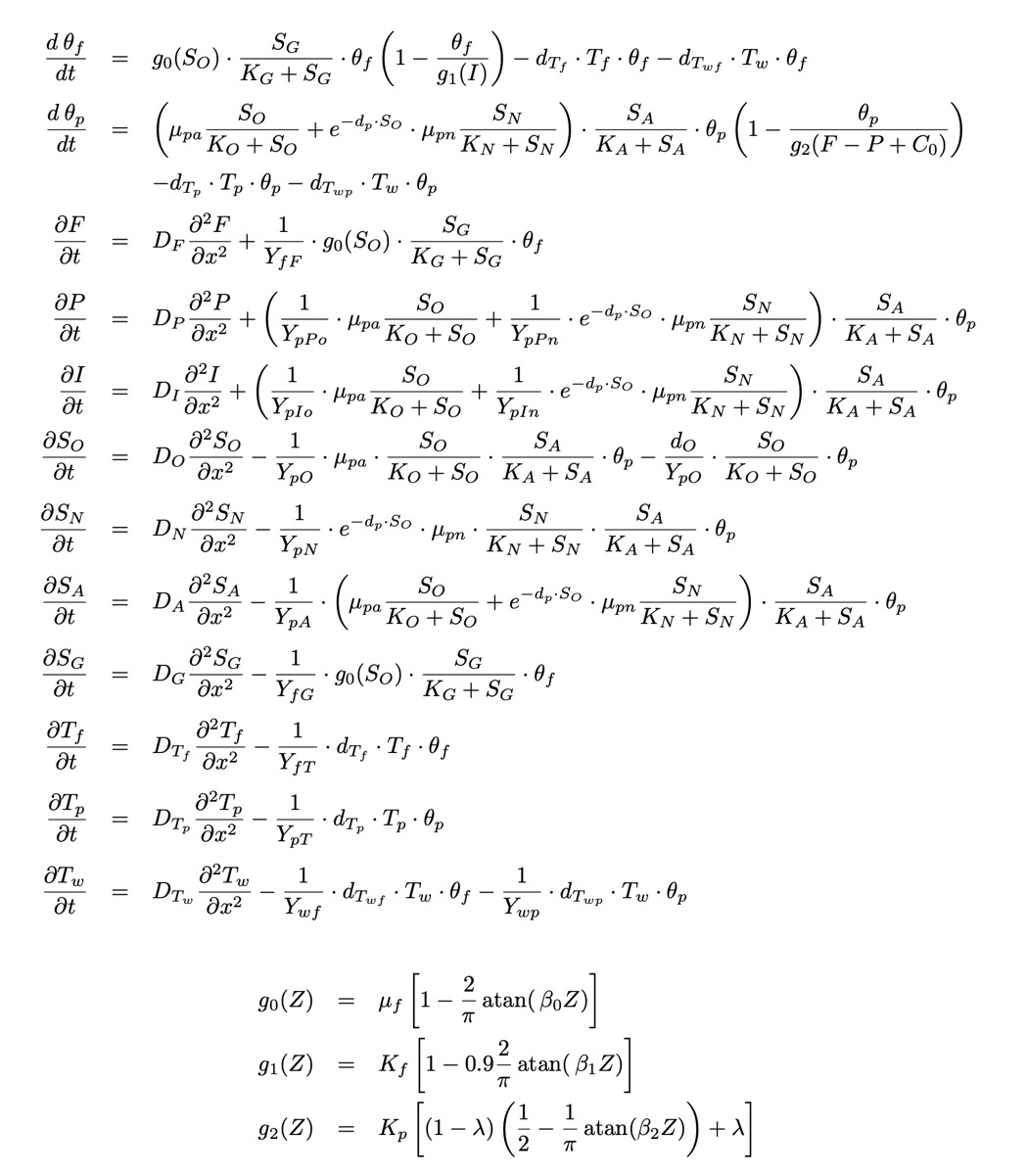
**

## Model Description and methodology

The model is defined on a spatial domain x [0 ∈ 0. 8] cm and consists of (i) two ordinary differential equations (ODEs) (in time) for the bacteria populations, with spatial variable as a parameter, and also (ii) ten reaction-diffusion partial differential equations (PDEs) for chemical species concentration, with no-flux boundary conditions at both boundaries except oxygen, which take fixed values at the top boundary x = 0.8 cm. The model equations are featured in the supplemental material.

Quantities θ_f_ (x, t) and θ_p_(x, t) are, respectively, measures of the fermenter and pathogen populations. Chemical species concentrations are denoted by S_O_ (oxygen), S_N_ (nitrate), S_G_ (sugar), S_A_ (amino acid), P (ammonium), F (acid), and I (inhibitor). In addition, three diffeent antibiotic concentrations are introduced: T_f_ which kills only fermenters, T_p_ which only kills Pathogen, and T_w_ which kills both. Fermenters (θ_f_ ) consume sugar (S_G_) and produce acid (F ). Pathogen consumes oxygen (S_O_) and amino acids (S_A_), and produces ammonium (P ) and a chemical inhibitor of fermenters (I). The growth rate of fermenters decreases with increasing oxygen concentration (through function g_0_), and the carrying capacity of fermenters is a decreasing function (g_1_) of I, reflecting that the presence of Pathogen inhibits fermenter growth. Likewise, the carrying capacity of Pathogen is a decreasing function g_2_ of F − P + C_0_, reflecting improved growth at higher pH. Here C_0_ describes the initial buffering pH; the bigger C_0_, the smaller the pH. Antibiotics act to kill microbes. We assume that antibiotics are consumed in the process of killing bacteria. In particular, effect of pH on Pathogen is introduced into the carrying capacity through function g_2_(Z), where Z is a proxy for acid. Note limZ→∞ g(Z) = λK_p_ and limZ→ −∞ g(Z) = K_p_. Thus the parameter λ tunes the inhibition effect of the fermenters on Pathogen. Smaller λ means stronger inhibition.

Standard finite difference methods are employed to solve the reaction-diffusion equations with the spatial domain [0, 0.8] discretized into n subintervals of size ∆x = 1/n, (n = 100 in the computations shown here). The diffusion term was discretized by central difference scheme and treated implicitly in time. The linear reaction terms (such as for T_f_ , T_p_, T_w_) were treated implicitly in time, and the nonlinear Monod-kinetic terms (such as for S_O_, S_N_ , S_A_, S_G_) were treated half implicit (the numerator term) and half explicit (the denominator term) in time. The ODEs for θ_f_ , θ_p_ were solved by the explicit fourth order four-stage Runge-Kutta method.

In our simulation, oxygen are supplied from the top of the tube; all other chemicals have no external source or sink. In order to compare with experimental data, our model takes the relative abundance data of pathogens and fermenters as input (initial value for the pathogen population θ_p_ and fermenters population θ_f_ uniformly distributed in the reactor). For each given input, one control with no antibiotics (NT) and three different antibiotic treatment strategies with T_f_ , T_p_, T_w_ are applied. The total amount of pathogens and fermenters at the end of the 50 hours treatment (integration of θ_p_  and θ_f_ over the spatial domain) are calculated and also converted to relative abundance for comparison with experimental results. We used the sputum microbiome data of all 24 patients as inputs for the model. In our analysis, we performed two iterations of the model, with the parameter λ in the function g_2_(Z) (reflecting the inhibition of fermenters on pathogen growth) being 0.1 and 0.05 respectively. Since smaller λ means stronger inhibition, iteration 1 (λ = 0.1) simulates weak inhibition on pathogen and iteration 2 (λ = 0.05) simulates strong inhibition on pathogen. Table S2 gives the parameter values used in the simulation, where parameters without units are dimensionless. Definitions of parameters are given in the caption of table S2. Figure S1 shows the total biomass ratio of Tf and NT treatments for patient 12 as a function of λ, where λ ranges from 0.01 to 0.2, with a 0.01 increment. It shows that as λ decreases (inhibition becomes stronger), the total biomass increases more under Tf treatment in comparison to the NT case.

# Supplementary Figures and captions

**
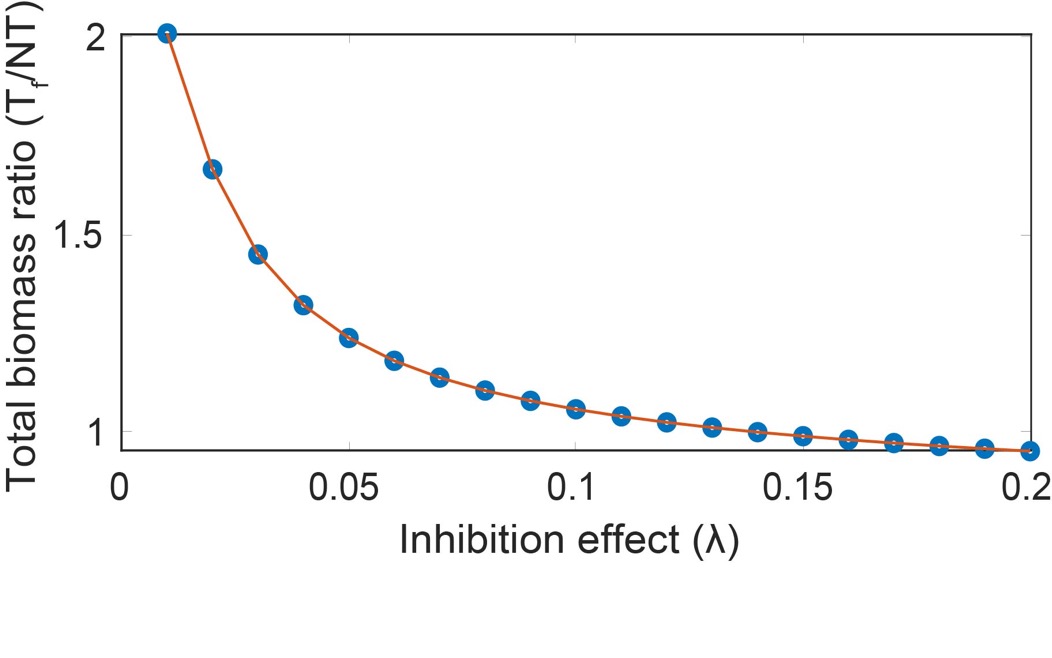
**

Figure S1. Total biomass ratio of Tf to NT treatment. Illustrated is inhibition effect of lambda on the total biomass between the no treatment (NT) and fermenter targeting antibiotic T_f_ within a singular patient 12.

**
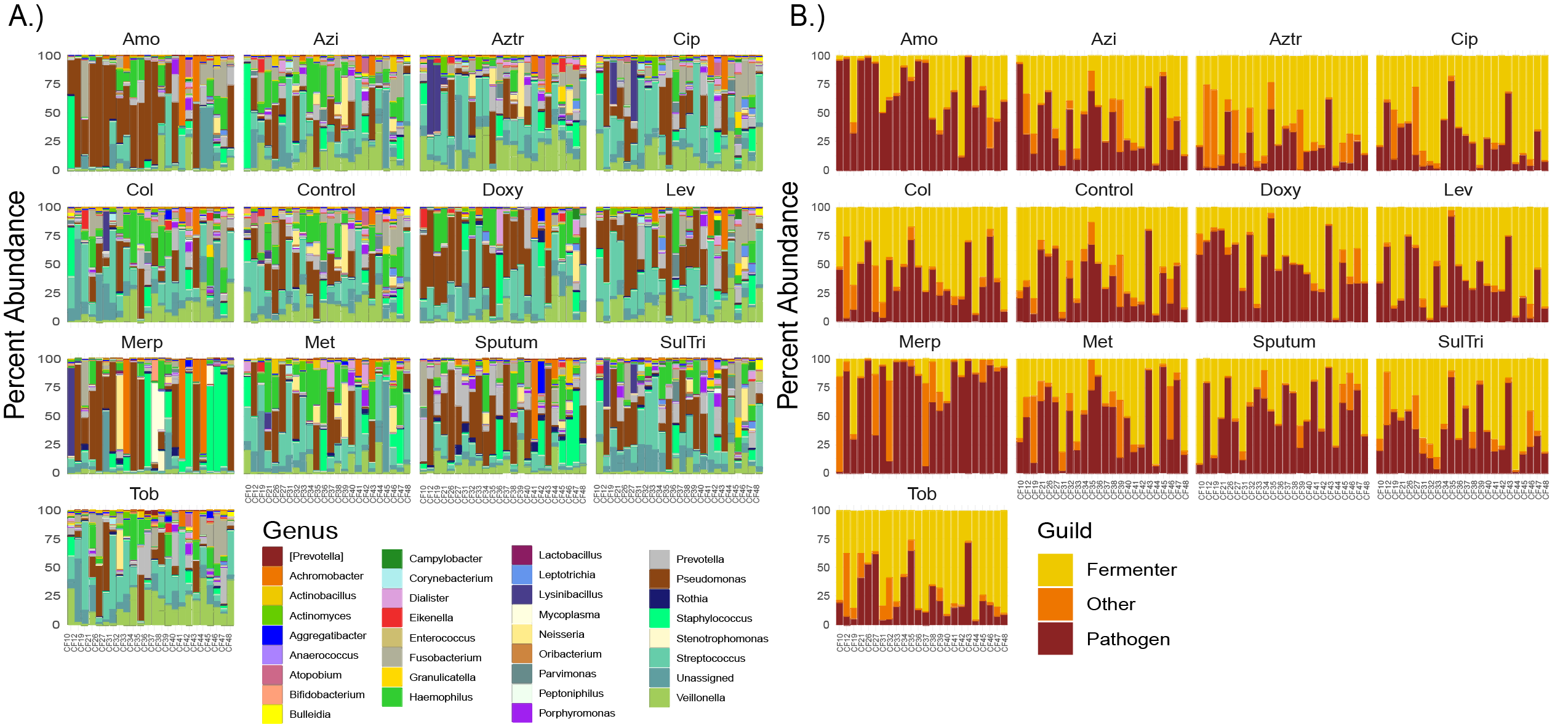
**

Fig. S2. Distribution of Microbial taxa and types. (A) Genera distribution across patients within each antibiotic patient. (B) Distributions based on genera-classification as classical pathogen or anaerobic fermenter, across patients, within each antibiotic treatment.


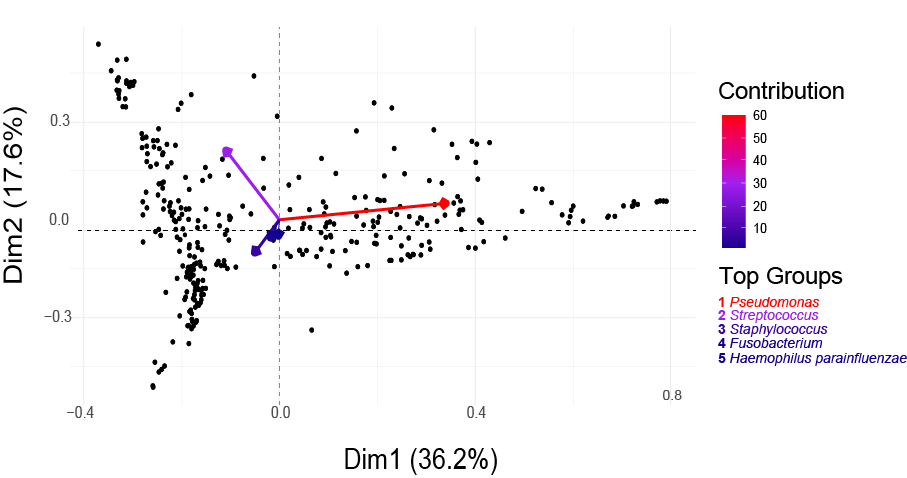


Fig. S3. PCA biplot. Illustrated are the top five organisms that drive microbiome differences within the samples. Arrows are each group driving change. Red is high percent contribution and blue is low percent contribution.


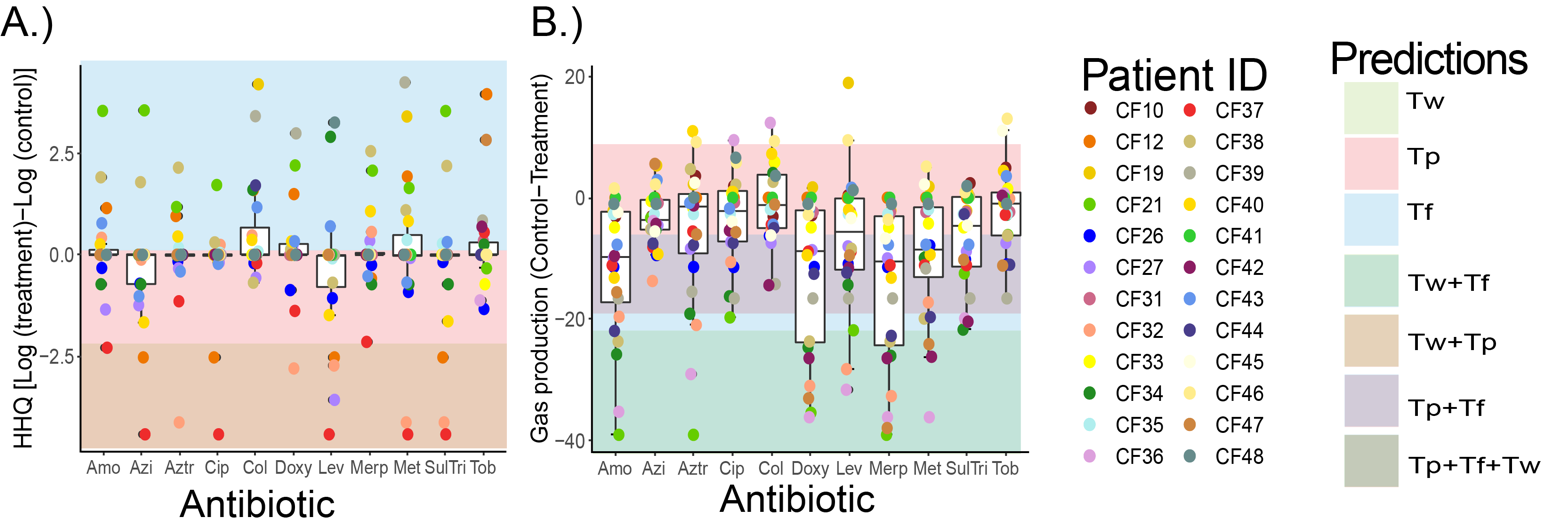


Figure S4. Results of overall community change compared to the control. The impacts of antibiotics (n=11) on (A) HHQ log difference and (B) gas production difference. Individual points are colored by patient (n=24) and predicted ranges are highlighted in each of the panels according to antibiotic treatment type (Tw, Tp, and Tf) to the control (NT) are also listed. Kruskal-Wallis statistics are reported in Table S3. Mann-Whitney post hoc tests are reported in Tables S13 and S15.


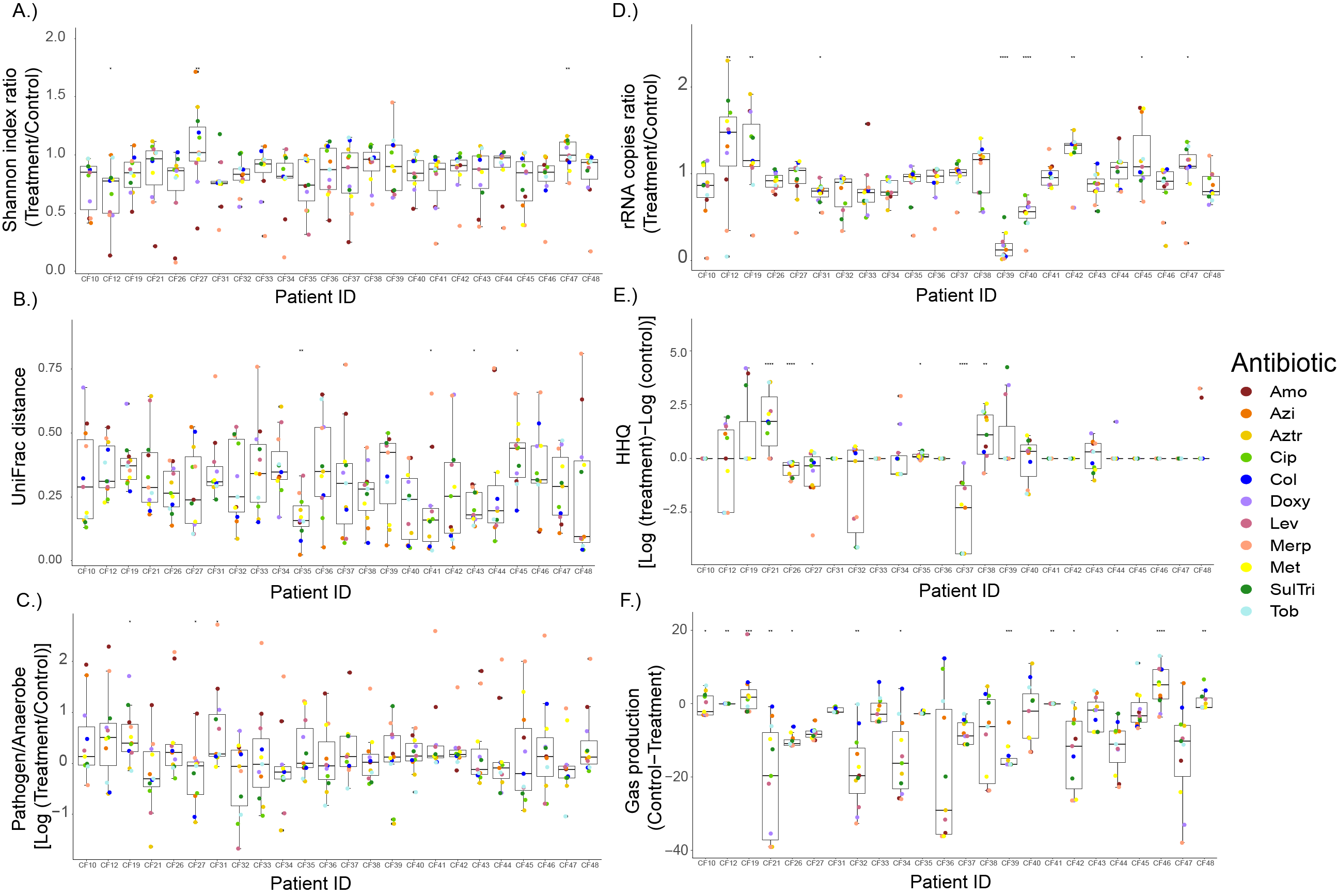


Figure S5. Results of overall community change compared to the control. The impact of individual patient (n=24) on (A) Shannon index ratio, (B) Weighted UniFrac distance, (C) pathogen to fermenter log ratio, (D) rRNA copy ratio, (E) HHQ log difference, and (F) gas production difference. Individual points are colored by antibiotic treatment (n=11). Kruskal-Wallis statistics are reported in Table S8.


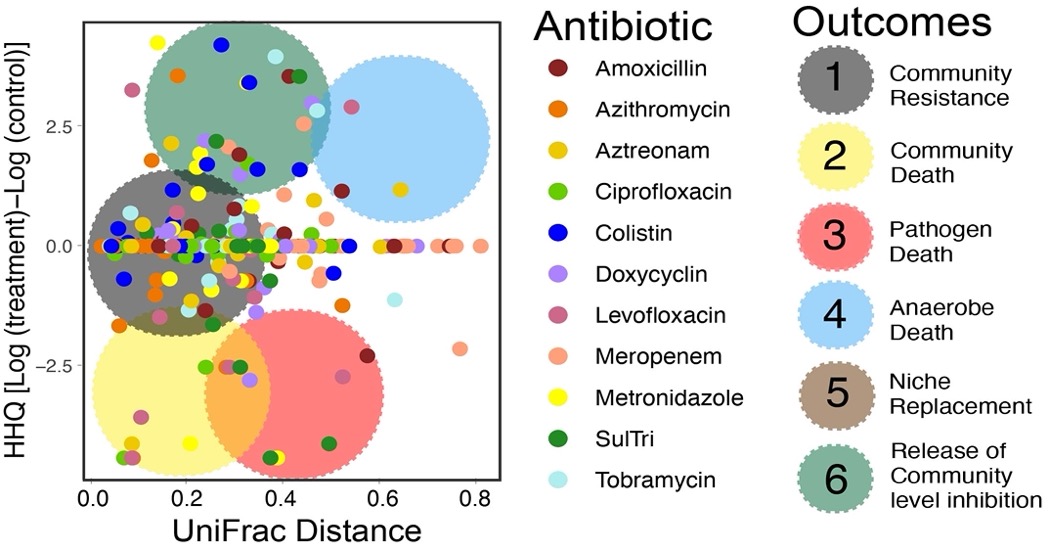


Figure S6. Community changes across various measures. Weighted UniFrac distance compared to HHQ abundance. Predicted outcomes (Community resistance, community death, pathogen death, anaerobe death, niche replacement, and release of community level inhibition) are indicated via circles on each of the panels.


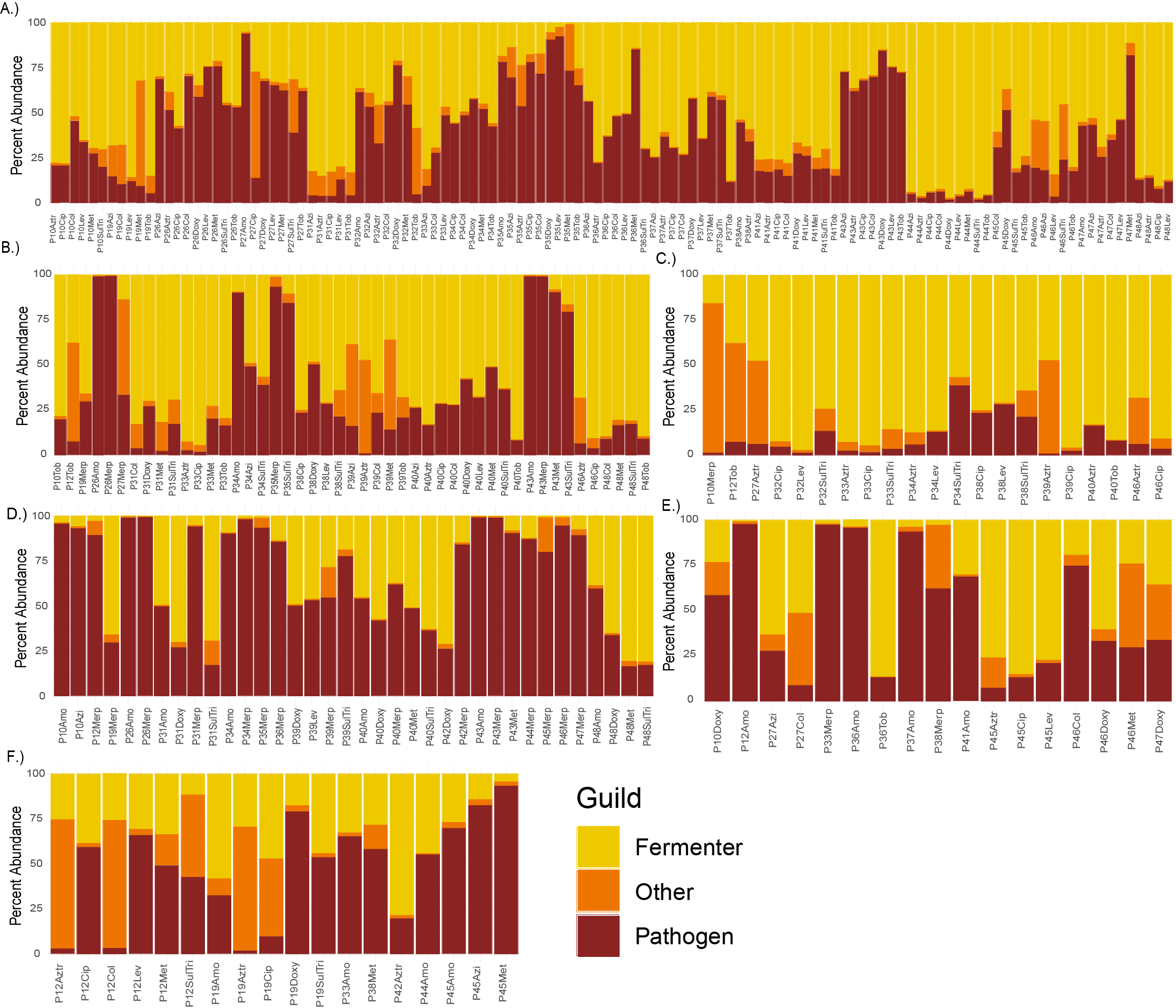


Figure S7. Microbiome of all Outcomes. The taxa of the microbiome were categorized into general types: anaerobe, pathogen, or other based on tables S2 and S3. The percent abundance of each of the outcomes are as follows: (A) community resistance, (B) community death, (C) pathogen death, (D) anaerobe death, (E) niche replacement and (F) release of community level inhibition.

**
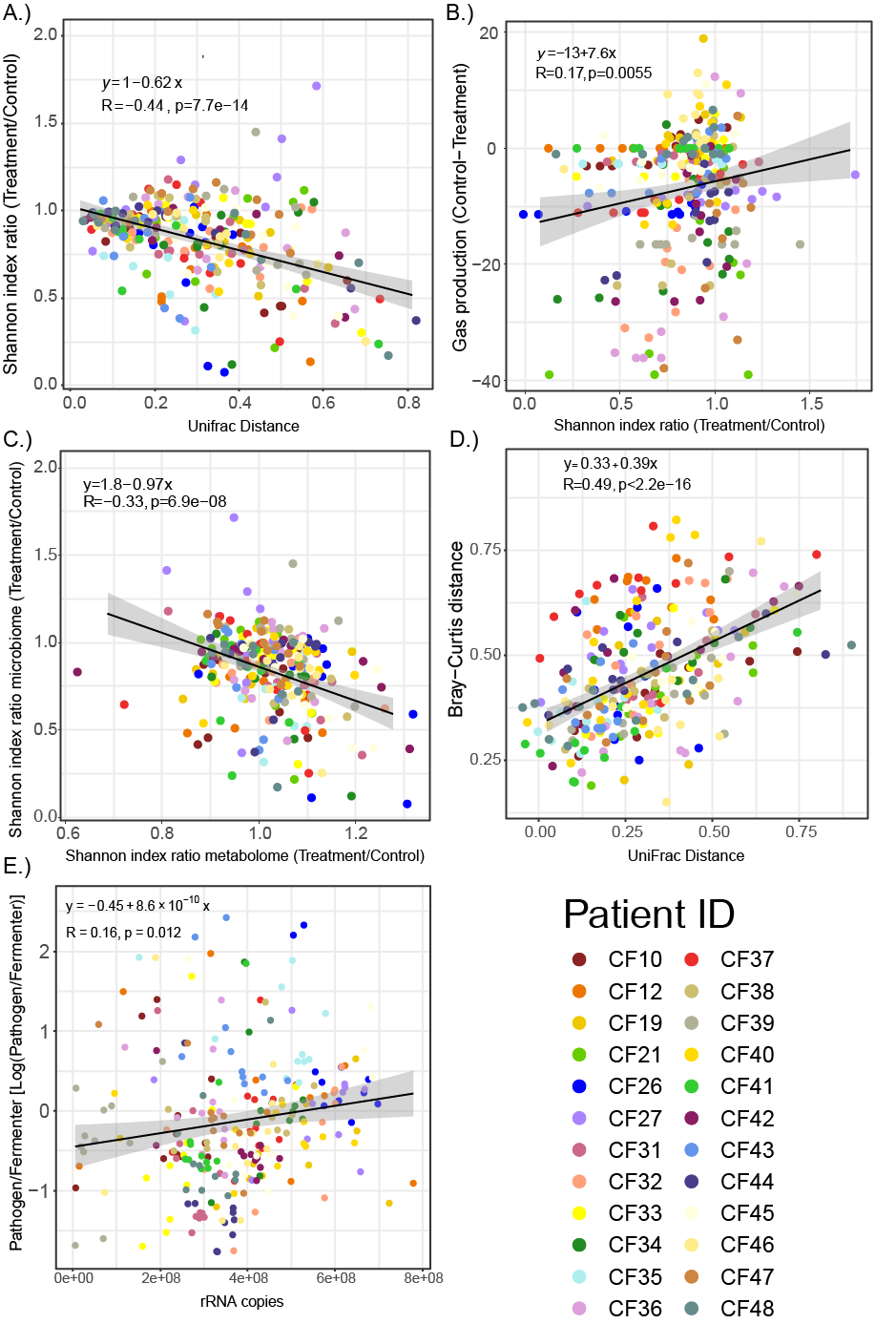
**

Figure S8. Comparisons across various measures with linear regressions. The following measures along with plotted linear regression equation, R values, and p values: (A) Shannon index of the microbiome versus UniFrac distance (compared to their respective controls), (B) gas productions versus Shannon index of the microbiome (compared to their respective controls), (C) Shannon indices of the microbiome and the metabolome (compared to their respective controls), (D) Bray-Curtis distance of the metabolome versus the UniFrac distance (compared to their respective controls) and (E) Pathogen/Fermenter log-ratio versus the number of rRNA copies.


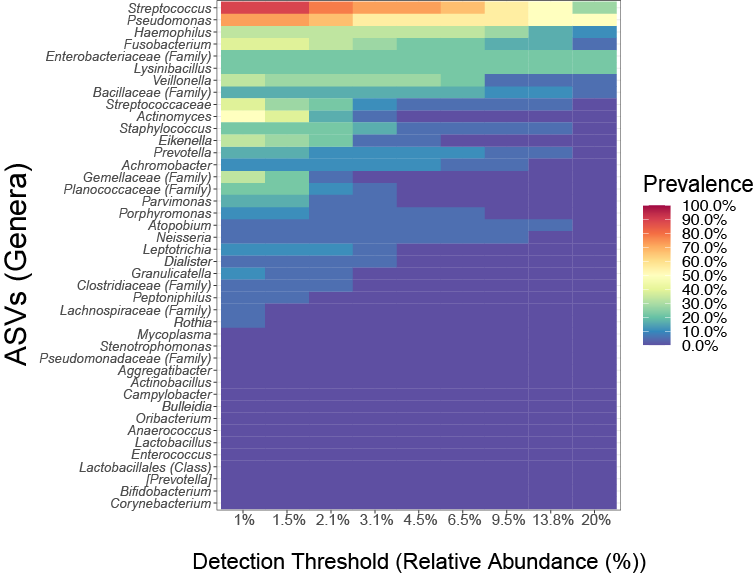


**Figure S9. Heatmap of prevalence measures**. We calculated the prevalence of ASVs using the relative abundances of the samples that had a 40% increase in UniFrac distance.


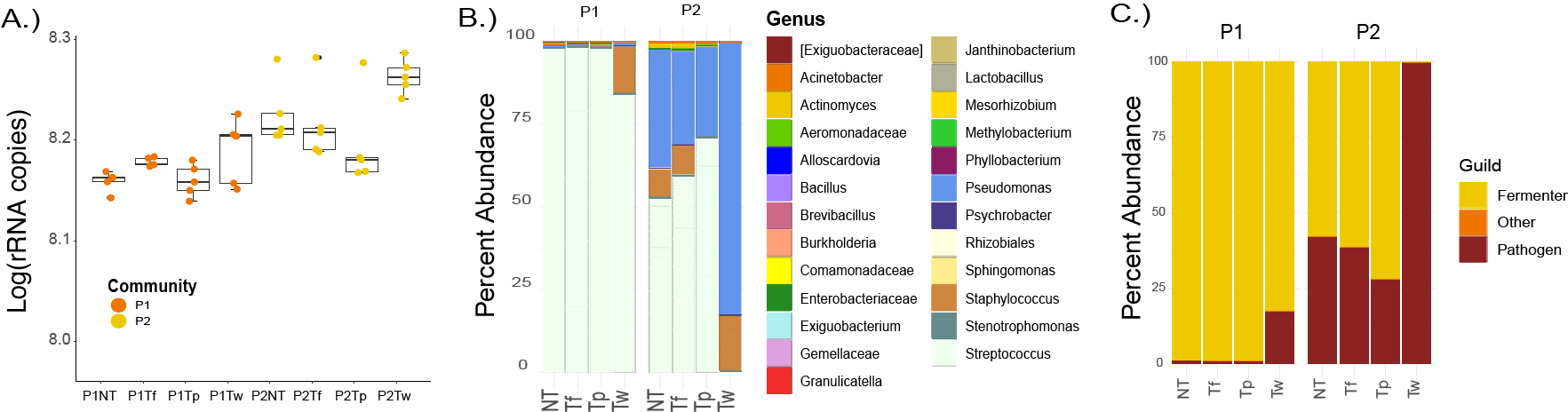


Figure S10. Model verification with five replicates. Subsequent validation using two experimental communities, P1 and P2, showing the (A) Log rRNA copies, (B) Genera abundance and (C) general bacterial type distribution.

# Supplementary Table Captions

Table S1. In vitro antibiotic information. Name, abbreviation, antibiotic class, solubility, concentration, equivalent model drug, and description are all listed with references.

**Table S2. Parameter used in both model iterations.** Numerical parameters are listed by the symbol, numerical value, and unit. D_F_, D_P_, D_I_, D_O_, D_N_, D_A_, D_G_, D_Tf_, D_Tw_, and D_Tp_ represents the diffusion coefficients of all the chemicals and antibioitcs with subscripts indicating the types of chemicals of antibioitcs. d_Tf_, d_Tw_, and d_Tp_ is the killing rate of T_f_, T_p_ and T_w_ on both θ_p_ and θf. μ_pa_, μ_pn_, μ_f_ are the maximum specific growth rates of pathogens under aerobic and anaerobic conditions, and maximum specific growth rate of the fermenters. d_O_ is the oxygen consumption rate by endogenous respiration of pathogens. K_f_ and K_p_ are the carrying capacity for fermenters and pathogens. d_p_ is the inhibition parameter on anaerobic respiration of pathogens by oxygen. K_G_, K_O_, K_N_, K_A_ are half saturation constants of Monod-kinetics for various chemicals. Y_fF_, Y_pPo_, Y_pPn_, Y_pIo_, Y_pIn_, Y_pO_, YpN, Y_pA_, Y_fG_, Y_fT_, Y_pT_, Y_wf_, and Y_wp_ are various yield coefficients characterizing the relation between growth of biomass and consumption/production of chemicals e.g. Y_pA_ is the production of biomass per unit consumption of amino acid. β_0_, β_1_, β_2_ are dimensionless parameters in functions defining the growth rate of fermenters and carrying capacity of both fermenters and pathogens. NA indicates not applicable.

**Table S3- List of pathogens (16S rRNA).** Taxon categorized as pathogens according to current CF literature.

**Table S4-List of pathogens (next-generation sequencing).** Taxon categorized as pathogens according to current CF literature.

**Table S5- List of Fermenters (16S rRNA).** Taxon categorized as fermenters according to current CF literature.

**Table S6- List of Fermenters (next-generation sequencing).** Taxon categorized as fermenters according to current CF literature.

**Table S7- PERMANOVA values.** The effects of antibiotic only, individual patient only, and combined effects of patients and antibiotic treatments were tested. P values < 0.05 are considered significant.

**Table S8-Kruskal-Wallis results.** A Kruskal-Wallis test was run per each measurement type against antibiotic treatment. P values < 0.05 are considered significant.

**Table S9-Kruskal-Wallis results.** A Kruskal-Wallis test was run per each measurement type against patient. P values < 0.05 are considered significant.

**Table S10-Mann-Whitney post hoc test for Shannon diversity indices.** A post hoc Mann-Whitney test was run to compare Shannon diversity indices compared to the control against each antibiotic. P values < 0.05 are considered significant and are highlighted in yellow.

**Table S11-Mann-Whitney post hoc test for weighted UniFrac distance.** A post hoc Mann-Whitney test was run to compare weighted UniFrac distances compared to the control against each antibiotic. P values < 0.05 are considered significant and are highlighted in yellow.

**Table S12-Mann-Whitney post hoc test for Pathogen to Fermenter ratio.** A post hoc Mann-Whitney test was run to compare the Log Pathogen to Fermenter ratio compared to the control against each antibiotic. P values < 0.05 are considered significant and are highlighted in yellow.

**Table S13-Mann-Whitney post hoc test for TBL (rRNA copies).** A post hoc Mann-Whitney test was run to compare rRNA copy ratio compared to the control against each antibiotic. P values < 0.05 are considered significant and are highlighted in yellow.

**Table S14- Mann-Whitney post hoc test for HHQ abundance by antibiotic.** A post hoc Mann-Whitney test was run to compare HHQ Log difference against each antibiotic. P values < 0.05 are considered significant.

**Table S15- Mann-Whitney post hoc for HHQ abundance by patient.** A post hoc Mann-Whitney test was run to compare HHQ Log differences against each patient. P values < 0.05 are considered significant and are highlighted in yellow.

**Table S16-Mann-Whiney post hoc test for Gas production.** A post hoc Mann-Whitney test was run to compare differences in gas production against each antibiotic. P values < 0.05 are considered significant and are highlighted in yellow.

**Table S17-Outcome categorization of samples.** Cutoff values were assigned based on the outcomes outlined in Figure 1 and Figure 2. Sputum and control samples are not included in this calculation.

**Table S18- List of Samples in Outcome 6.** Samples that fall under the cutoff values outlined in table S16.

**Table S19.** **Patient information (next generation sequencing samples).** Patient ID, sample type (i.e patient status), days between treatment, gender, FEV1, a current medications are listed here. NA indicated not applicable

**Table S20. Sputum sample information by patient for experimental results.** Patient ID, age, gender FEV1, FVC, Clinical microbiology, patient status, and current antibiotics (oral, IV and inhaled) are listed here.

**Supplemental Material References**

1. Ameen SM, Rolain JM, Le Poullain MN, Roux V, Raoult D, Drancourt M. Serum concentration of co-trimoxazole during a high-dosage regimen. *J Antimicrob Chemother* 2014; **69**: 757–760.

2. Wilms EB, Touw DJ, Heijerman HGM. Pharmacokinetics and sputum penetration of azithromycin during once weekly dosing in cystic fibrosis patients. *J Cyst Fibros* 2008; **7**: 79–84.

3. Sherrard LJ, Graham KA, McGrath SJ, McIlreavey L, Hatch J, Muhlebach MS, et al. Antibiotic resistance in prevotella species isolated from patients with cystic fibrosis. *J Antimicrob Chemother* 2013; **68**: 2369–2374.

4. Delfino E, Fucile C, Del Bono V, Marchese A, Marini V, Coppo E, et al. Pharmacokinetics of high-dose extended-infusion meropenem during pulmonary exacerbation in adult cystic fibrosis patients: A case series. *New Microbiol* 2018; **41**: 47–51.

5. de Velde F, de Winter BCM, Koch BCP, van Gelder T, Mouton JW. Non-linear absorption pharmacokinetics of amoxicillin: Consequences for dosing regimens and clinical breakpoints. *J Antimicrob Chemother* 2016; **71**: 2909–2917.

6. Gibson EK, McKay DS, Thomas-Keprta KL, Wentworth SJ, Westall F, Steele A, et al. Life on Mars: Evaluation of the evidence within Martian meteorites ALH84001, Nakhla, and Shergotty. *Precambrian Res* 2001; **106**: 15–34.

7. Horrevorts AM, Degener JE, Dzoljic-Danilovic G, Michel MF, Kerrebijn KF, Driessen O, et al. Pharmacokinetics of tobramycin in patients with cystic fibrosis. Implications for the dosing interval. *Chest* 1985; **88**: 260–264.

8. Yapa SWS, Li J, Patel K, Wilson JW, Dooley MJ, George J, et al. Pulmonary and systemic pharmacokinetics of inhaled and intravenous colistin methanesulfonate in cystic fibrosis patients: Targeting advantage of inhalational administration. *Antimicrob Agents Chemother* 2014; **58**: 2570–2579.

9. Beringer PM, Owens H, Nguyen A, Benitez D, Rao A, D’Argenio DZ. Pharmacokinetics of doxycycline in adults with cystic fibrosis. *Antimicrob Agents Chemother* 2012; **56**: 70–74.

10. Dalhoff A. Pharmacokinetics and pharmacodynamics of aerosolized antibacterial agents in chronically infected cystic fibrosis patients. *Clin Microbiol Rev* 2014; **27**: 753–782.
